# Urban greenspace types and climate factors jointly drive the microbial community structure and co-occurrence network

**DOI:** 10.1038/s41598-024-66588-8

**Published:** 2024-07-11

**Authors:** Huan Wang, Yilong Feng, Qiaoyong Zhang, Min Zou, Ting Li, Lijiao Ai, Haiyang Wang

**Affiliations:** 1 College of Horticulture and Landscape Architecture, Southwest University, Chongqing, 400718 China; 2 Chongqing Landscape and Gardening Research Institute, Chongqing, 401329 China; 3 Chongqing Key Laboratory of Germplasm Innovation and Utilization of Native Plants, Chongqing, 401329 China

**Keywords:** Urban green space, Biodiversity, 16S ribosomal RNA, Phyllosphere bacteria, Bark epiphytic bacterial, Microbiology, Ecology

## Abstract

The benefits of urban green space are socially widely recognized as a direct link between plant–microbe interactions and the maintenance of biodiversity, community stability, and ecosystem functioning. Nevertheless, there is a lack of knowledge about the factors influencing microbial communities in urban green spaces, especially those related to phyllosphere epiphytes and stem epiphytes. In this study, we analyzed the microbial community assembly in leaf and stem bark samples collected from Square, Road, Campus, and Park. Illumina sequecing of 16S amplicons was performed to characterize microbial diversity and composition. The α-diversity was significantly higher in the bark epiphytic community, compared to the phyllosphere. Moreover, urban greenspaces'type altered the way communities gathered. The main soil and air properties factors of the urban greenhouse (e.g. soil temperature, atmospheric moisture, air temperature) were shaping the characteristics of bacterial communities on the leaf surface and bark epiphytic. In addition, in the co-occurrence network analysis, keystone taxa were not mostly observed in abundant species, which may be necessary to maintain ecosystem functions. Finally, our findings provide a deeper understanding of the ecological dynamics and microbial interactions within plant phyllosphere and stem epiphytes microbiomes.

## Introduction

Due to the growing urbanization of human populations, green spaces have become increasingly important for human health^1,2^. The evidence for the health benefits of urban green spaces biodiversity is growing, including reduced blood pressure, reduced pain, lower cortisol levels, and reduced mortality rates from all causes^3–5^. In the urban ecosystem, microorganisms play an essential role in regulating ecosystem services^6,7^, such as pollutant remediation, nutrient cycling, and genetic diversity conservation^8^. Urban ecosystems must perform these functions to reduce the growing global burden of chronic diseases associated with urban environments and to maintain the well-being of citizens living in cities. Human health can be compromised by the loss of contact with a diverse environment microbiome (a specific environment's community of microbes, including bacteria, fungi, archaea, and viruses), as well as key microbial taxa—microbial 'old friends'^9,10^. Experiencing environmental microbiomes shapes the human microbiome, which helps educate and maintain a healthy immune system^11,12^. It directly influences the development of the immune system^13^ and predisposition to infectious and non-infectious diseases^14,15^. Human exposure to soil and plant microorganisms in urban green spaces appears to suppress inflammation and reduce immune dysfunction^16,17^. Despite the lack of understanding about why these positive health outcomes occur, it is likely a result of human interactions with animals, plants, and microbes.

Land uses have changed dramatically over the past several decades due to the massive shift of population from rural to urban areas, potentially changing microorganism habitats^18^. To predict and mitigate the impact of anthropogenic disturbances on microbial communities in urban environments, we must have a better understanding of how these disturbances affect them^19,20^. While urban microbial ecosystems are likely to play an important role in human health, they are not well studied in comparison to animal and plant communities^21^.

Management practices are intensive in urban ecosystems, The microbial community in urban green spaces is influenced not only by the environment (Factors such as soil properties and climate), but also by human interactions^6,22^. To date, a few studies have reported that urbanization can substantially change the microbiota in urban green spaces^22,23^, In urban green spaces, however, there is a great deal of variation in microbial community profiles, and it remains unclear what factors contribute to this. The impact of urbanization on soil bacteria has largely been studied. The phyllosphere plays a significant role in microorganisms' habitat, but the phyllosphere and bark epiphytic bacteria have been overlooked.

According to previous studies, the phyllosphere provides a great opportunity to test basic ecological principles in microbiology^24^. With an estimated area of more than 1 billion square kilometers, it is one of the world's largest environments^25,26^. The phyllosphere microbiome plays a variety of important ecosystem functions besides supporting plant growth^27^. Other ecosystem functions are performed by phyllosphere microbiomes, for example, reducing plant ethanol emissions and fixing nitrogen as part of Earth's biogeochemical cycles^28^. Also, as phyllosphere microbiomes serve as a bridge between environmental and human microbiomes, phyllosphere microbiomes are closely related to human health^29^. Community composition and diversity of epiphytic species are affected not only by air pollution, but also by growth habitat, tree characteristics (e.g., bark properties such as water-holding capacity and bark pH)^30^, tree species^31^, etc.

The purpose of this study was to examine patterns of phyllosphere and bark epiphytic bacteria of different plants from urban green spaces in Yongchuan district, Chongqing, China. In our study, we examined bacterial abundance and diversity by high-throughput amplicon sequencing of the small-subunit ribosomal RNA (16S rRNA) gene. In addition, we examine well-replicated plots of four different types of urban green space to integrate these bacterial results with vegetation and environmental surveys (road green space, park green space, square green space, and campus green space). In our analysis, the main aims were to (i) determine the potential differences and links between leaf and bark phyllosphere bacterial communities; (ii) determine the most important factors shaping the bacterial community profiles in urban green spaces, and (iii) understanding community correlation network structure and keystone taxa of samples.

## Material and methods

### Sampling and experiment design

**Figure 1:**
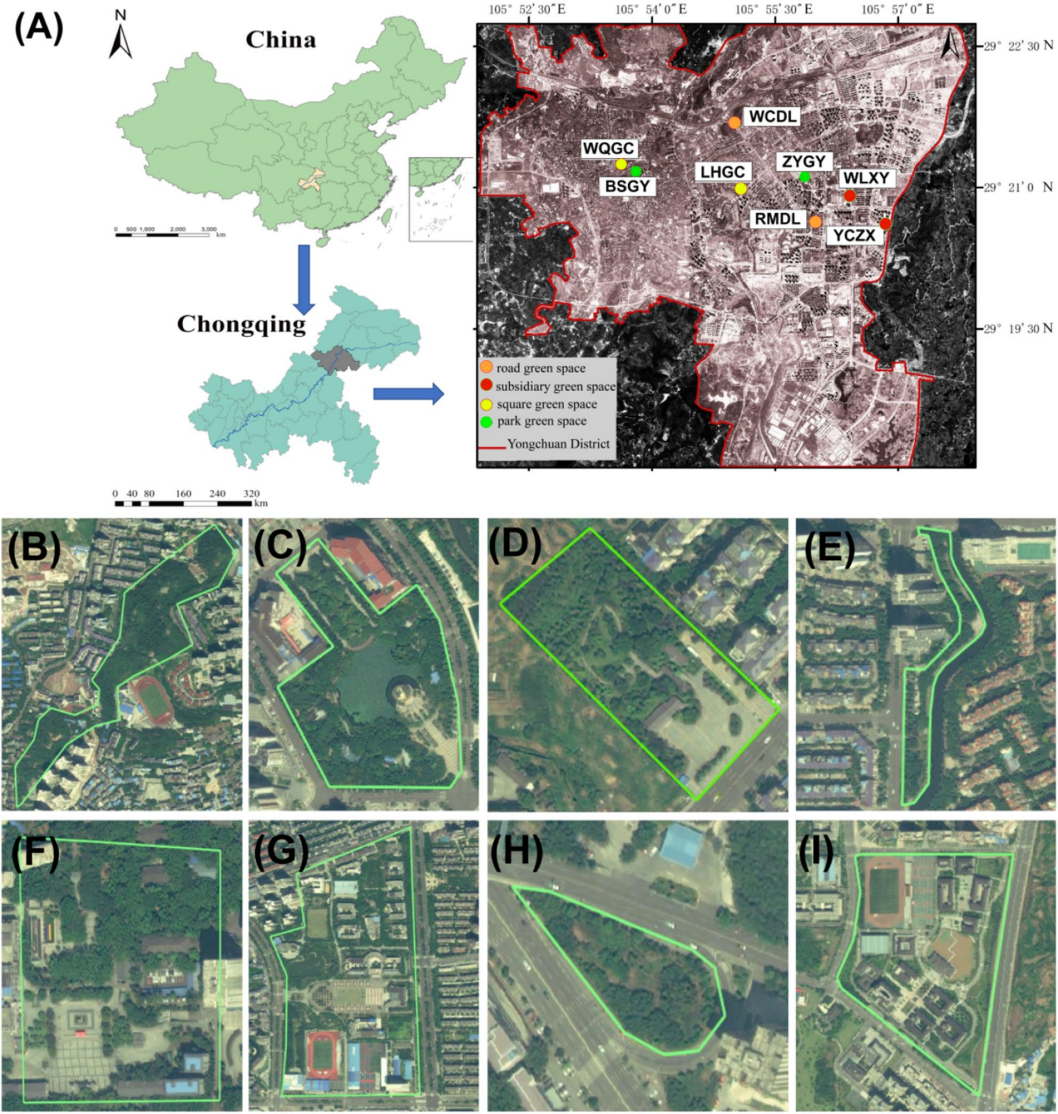
Sample sites information. (**A**) Sampling distribution of Yongchuan District, Chongqing City. (**B**–**I**) Satellite view of the site of BSGY, ZYGY, LHGC, WQGC, WCDL, RMDL, WLXY, YCZX espectively.

In April 2023, leaf and bark samples were collected at 8 sites in Yongchuan (105°93′ E, 29°36′ N), Chongqing, China. The sites included road greenspaces (2 sites), parks greenspaces (2 sites), square greenspaces (2 sites), and subsidiary community greenspaces (2 sites), which represent the typical types of greenspaces in urban environments (Fig. 1). Square green space is a venue for urban public activities with functions such as recreation, commemoration, assembly, and refuge. Park green spaces meet the leisure needs of urban residents and provide places for rest, excursions, exercise and other collective cultural activities. Road green space refers to the ground used for the cultivation of plants and landscaping within the land area of a road. It is mainly used to purify the air, beautify the environment, prevent noise, and guide the sight of drivers. Campus green space, as an adjunct to the school, provides a comfortable recreational and landscaped site for students and faculty. In order to minimize the effects of weather on sample data, sampling was conducted within one day after seven days without rainfall. In each sampling site, 10 × 10 m squared (m^2^) were constructed. Leaf and trunk epidermis samples were collected from dominant species of trees and dominant species of shrubs in different types of green spaces. Sterile scissors were used to collect leaves and trunk epidermis from three plants at the same stage of growth and from the same height above ground, and then the leaves and trunk epidermis were placed in labeled sterile bags. Collection of plant material comply with relevant institutional, national, and international guidelines and legislation. The basic information on the sampling points is shown in Table 1. Three parallel replicates were measured for each sample and a total of 90 samples were collected. The leaves and trunk epidermis samples were cut with sterilized scissors to obtain equal weight, placed in a sterile centrifuge tube with PBS buffer for a while, centrifuged to remove the supernatant, resuspended by adding 1 mL of buffer, snap-frozen in liquid nitrogen, and stored at − 80 °C for DNA extraction. Data of atmospheric concentration of meteorological parameters and gaseous pollutants (including wind speed, wind direction, atmospheric temperature, soil temperature, TBQ total radiation, radiation accumulation, soil humidity, atmospheric humidity, negative oxygen ions, noise, PM2.5, PM10) were obtained from the hourly monitor by atmospheric fixed-site monitoring stations.

**Table 1:** Information table of plots of different green space types.

ID	Type of green spaces	Sample Site	Coordinate		Altitude (m)	Area (hm^2^)	Tree dominant species	Shrub dominant species
			Longitude	Latitude				
1	Square green space	LHGC	105°54′52.72″	29°21′ 10.55″	314	1.29	*Magnolia denudata Desr*	*Alpinia zerumbet 'Variegata'*
2		WQGC	105°53′25.33″	29°21′26.95″	316	3.05	*Osmanthus fragrans*	*Aucuba japonica var. variegata*
3	Road green space	WCDL	105°54′49.57″	29°21′52.09″	320	0.65	*Bauhinia purpurea*	*Loropetalum chinense var. rubrum*
4		RMDL	105°55′45.32″	29°20′48.48″	304	1.61	*Magnolia grandiflora*	*Camellia japonica*
5	Campus Green space	WLXY	105°56′ 11.43″	29°21′4.73″	318	18.28	*Eucalyptus robusta*	*Spiraea salicifolia*
6		YCZX	105°56′39.33″	29°20′46. 12″	326	3.69	*Prunus cerasifera 'Atropurpurea'*	*Ligustrum lucidum*
7	Park green space	ZYGY	105°55′38.71″	29°21′ 17.46″	308	6.63	*Ficus virens*	*Pittosporum tobira*
8		BSGY	105°53′35.16″	29°21′20.59″	324	12.12	*Osmanthus fragrans*	

### DNA Extraction, PCR Amplification and Illumina MiSeq Sequencing

As directed by the manufacturer, genomic DNA was extracted from each sample using the E.Z.N.A.®Soil DNA Kit. Total DNA was eluted with 50 µL TE buffer (Tris-hydrochloride buffer, pH 8.0, 1.0 mM EDTA contained). In order to determine DNA concentration and purity, a NanoDropTM 1000 spectrophotometer (Thermo Fisher Scientific, Waltham, MA, USA) was used and samples were stored at − 80 °C until PCR amplification. PCR amplification of the V3–V4 region of the 16S ribosomal RNA gene was carried out on bacteria (98 °C for 30 s; with 35 cycles at 98 °C for 10 s, 54 °C for 30 s, and 72 °C for 45 s; and a final extension at 72 °C for 10 min) using a primer set of 341F (5′-CCTACGGGNGGCWGCAG-3′) and 805R (5′-GACTACHVGGGTATCTAATCC-3′)^32^. Barcodes were added to the 5′ ends of the primers and universal primers were used. Each PCR reaction was conducted in a 25 µL mixture containing 12.5 µL of 2 × Phusion® Hot Start Flex Master Mix, 2.5 µL of each primer (1 µM), and 50 ng of template DNA. Nuclease-free water served as blank. For the DNA extraction process, ultrapure water was used instead of a sample solution to ensure that there were no false positives. Throughout the DNA extraction process, ultrapure water was used in place of template DNA as a negative control to exclude false‐positive PCR results. 2% agarose gel electrophoresis was used to verify the PCR amplicon size. PCR products were purified using AMPure XT beads from Beckman Coulter Genomics in Danvers, MA, USA, and quantified using Qubit from Invitrogen, USA. The amplicon library was sized and quantified using an Agilent 2100 Bioanalyzer (Agilent, USA) and a Library Quantification Kit for Illumina (Kapa Biosciences, Woburn, MA, USA), respectively. A NovaSeq PE250 platform at LC-Bio Technology Company (Hangzhou, China) was used to sequence the libraries.

### Sequencing data processing

Based on the samples' unique barcodes, paired-end reads were assigned, then truncated by cutting off the barcodes and primer sequences, and merged with FLASH^33^. Fqtrim software was used to trim and filter the raw reads, and Vsearch software was used to further filter the chimeric reads^34^. The amplicon sequence variants (ASVs) were generated with the DADA2 package^35^. Through BLAST searches, all of the sequence reads were compared against the Silva rRNA database^36^, and the sequences clustered into OTU clustering at 100% similarity. Using the average abundance of each group, the relative abundance of each taxon was calculated by normalizing assigned reads to the total number of qualified reads. The rarefaction curves were generated using custom Perl scripts for each sample. The BioVenn software was used to plot Venn diagrams showing the shared and unique features (http://www.biovenn.nl/index.php accessed on 2 November 2022). To analyze the complexity of species, the alpha diversity indices (Observed species, Shannon diversity, and Simpson evenness) were calculated using QIIME 2 (Quantitative insights into microbial ecology 2)^37^.

### Statistical analysis

One-way ANOVA or t-tests was used to test the significance of variance between or among samples using SPSS 22.0 (SAS Institute Inc., Cary, NC, USA). An analysis of beta diversity was used to display and compare bacterial community compositions. With the QIIME 2 plugin, PCA (principal component analysis) was conducted^37^. Using the group average method, hierarchical agglomerative clustering (to group objects similar to each other in clusters) was carried out on the most abundant features according to the groups selected. By using the OmicStudio tool, a heatmap of bacterial communities was generated, with Bray–Curtis similarity calculations used to cluster relative abundance data^38^. Unless otherwise stated, all statistical analyses were conducted at a significance level of 0.05. With the "random Forest" and "rfPermute" R packages, we examined the most important environmental factors driving bacterial Shannon diversity^39^. Venn diagrams showing the shared and unique features were plotted, using BioVenn (http://www.biovenn.nl/index.php). Redundancy analysis was performed using the OmicStudio tools at https://www.omicstudio.cn/tool, which calculate of the correlation between environmental factors and the Bray–Curtis distance for microbial diversity in samples. Correlation network analysis used the igraph (Version 1.2.6) package of R (Version 3.6.3) based on the Spearman correlation.

## Result

### Sequence data results and alpha diversity of communities

**Figure 2:**
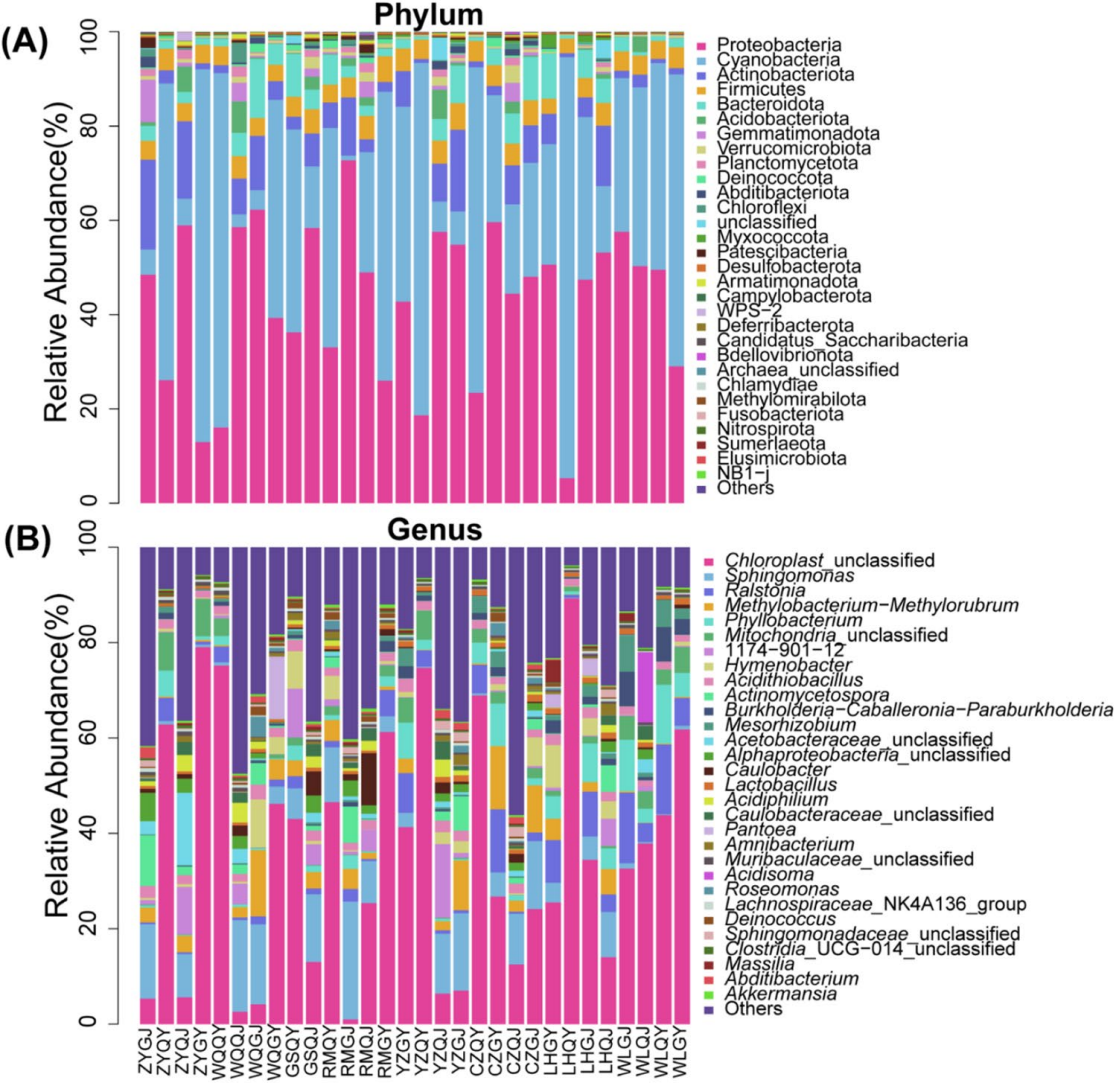
Relative abundance of different phyla (**a**) and genera (**b**) in the samples (top 30).

The sequencing process utilizing the 16S rRNA genes yielded a cumulative count of 1,892,424 sequences that underwent quality filtration, amounting to 1.23 Gb of valid data. The sequence count per sample varied between 38,366 and 72,275. Following dereplication using DADA2 within QIIME2, a total of 9,626 prokaryotic operational taxonomic units (OTUs) were acquired. As the quantity of sequencing data increased, all rarefaction curves exhibited saturation, resulting in an average Good's coverage of 88.93% across all samples (Supplementary Table S1). Proteobacteria (43.28%) were the most abundant phylum of bacteria, followed by Cyanobacteria (35.51%), Actinobacteriota (5.93%), and Firmicutes (4.37%). At the genus level, Chloroplast_unclassified (35.79%), Sphingomonas (7.28%), Ralstonia (4.11%), Methylobacterium-Methylorubrum (3.30%), and Phyllobacterium (3.20%) were the top 5 abundant genera, and these features accounted for 53.65% of the entire collection, while unclassified represents sequences marked as unclassified bacteria (Fig. 2).

### Species composition of bacterial communities among different content

**Figure 3:**
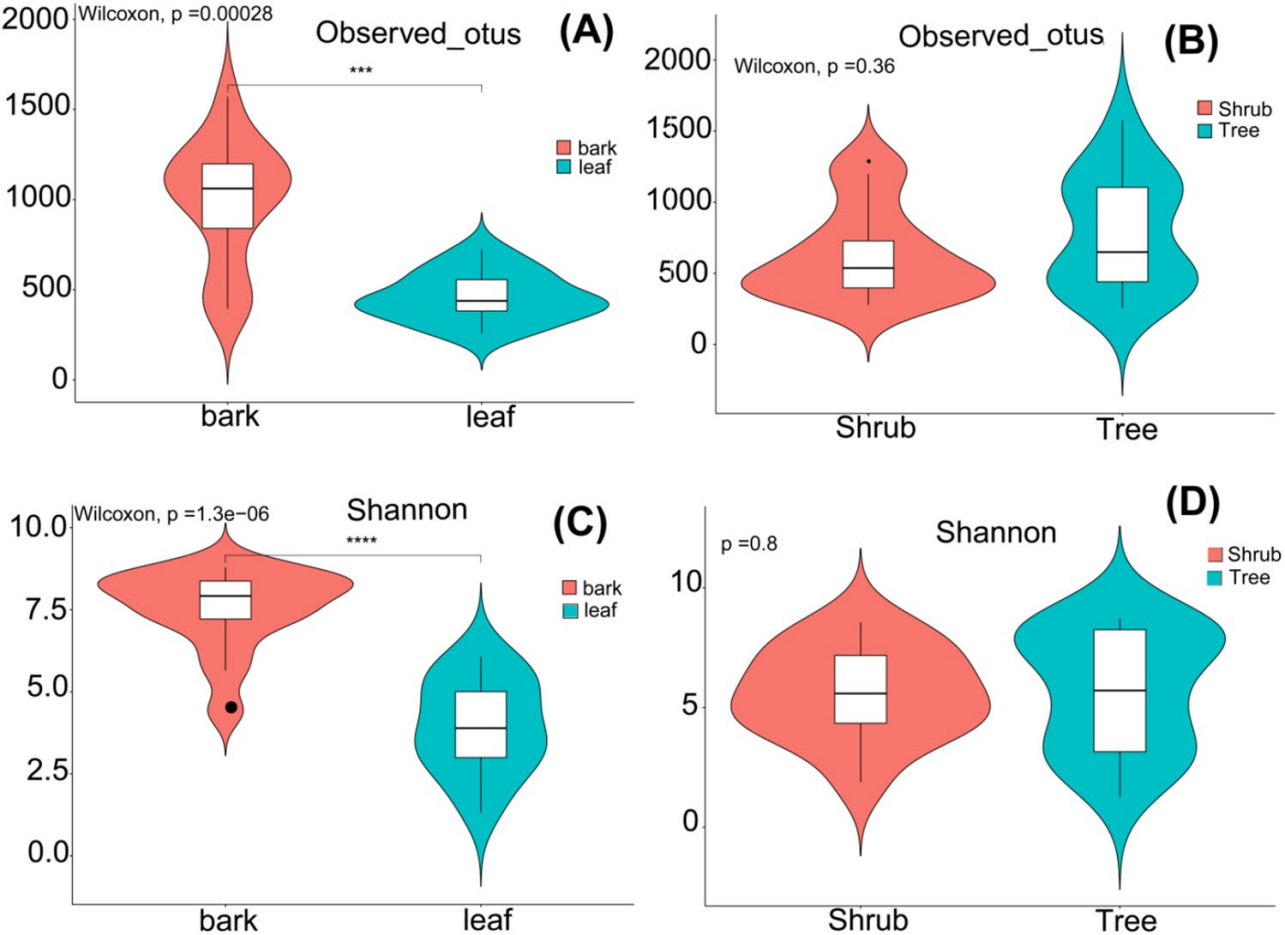
Violin plots displaying the alpha diversity measures (including the number of observed operational taxonomic units and Shannon diversity) for the bark vs leaf and shrub vs tree groups (**A**–**D**).

Comparing the bark group and leaf groups, there were significant disparities observed in the diversity indices (Shannon diversity and observed otus) (*p* < 0.05; Fig. 3A,C). Conversely, no statistically significant distinction was identified in the alpha diversity indices when comparing the shrub and tree groups (Shannon diversity and observed otus) (*p* > 0.05; Fig. 3B,D).

**Figure 4:**
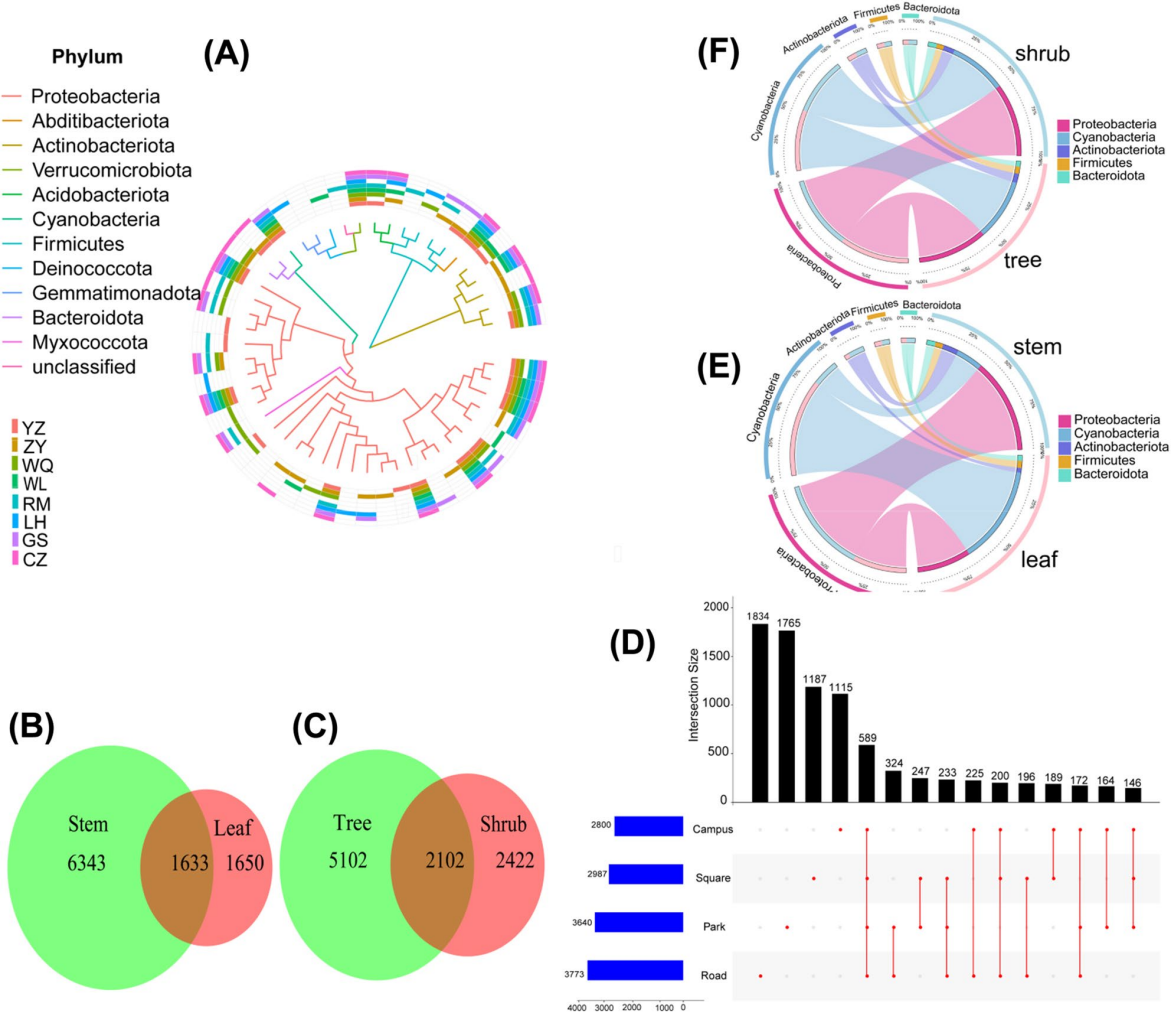
(**A**) Phylogenetic diversity of the bacterial (Unifrac distance matrices). (**B**, **C**) Venn diagram showing the numbers of unique and shared genera in bark and leaf, tree and shrub phyllosphere samples. (**D**) Upset plots showing variation partitioning models of 4 four urban green space community composition. (**E**, **F**) Circos plot of the top 5 abundant bacterial features at the phylum levels in bark and leaf, tree and shrub phyllosphere samples.

Regarding the discrepancies in bacterial characteristics between the stem and leaf groups, as depicted in a Venn diagram, it was observed that these two groups shared 1633 bacterial features in common, while the bark and leaf group possessed 6343 and 1650 unique features, respectively (Fig. 4B). Similarly, the tree and shrub groups exhibited 5102 and 2402 unique features, respectively, with 2102 bacterial features being common to both groups (Fig. 4C). In the within-group variation analysis of different green space types, road green space contained the most microbial species at 3773. This was followed by parks, plazas, and ancillary (campus) green space at 3640, 2987, and 2800, respectively. A total of 580 cooccurring types were found in several types (Fig. 4D).

From samples of all sites, the most abundant microbial phylum was Proteobacteria (43.28%), and other major groups were Cyanobacteria (35.51%), Actinobacteriota (5.93%), Firmicutes (4.37%) and Bacteroidota (4.19%) (Fig. 4A). Samples were analyzed for clustering using Bray–Curtis distance analysis. Among the different green space types, road green spaces (CZ and RM) had the closest clustering of sample composition and the most similar surface community structure, while the other three green spaces (square green space, campus green space, and park green space) types were not differentiated. The five most abundant phyla were consistent across the tree-shrub and bark-leaf groups, respectively. However, the proportions of distribution of each dominant species varied considerably between sample groups. Proteobacteria and Cyanobacteria accounted for 44.53% and 34.63% of the shrub group, respectively. Similarly, the dominant species in tree groups, Proteobacteria and Cyanobacteria accounted for 41.85% and 37.05% of the tree group, respectively (Fig. 4F). However, within the leaf and bark, there were large differences in the proportion of dominant species composition. The most two abundant microbial phylum in the bark were Proteobacteria (54.77%) and Cyanobacteria (15.55%). In the leaf group, the dominant species is Cyanobacteria rather than Proteobacteria, with the proportions of Cyanobacteria and Proteobacteria being 56.46% and 31.25%, respectively (Fig. 4E).

### Environmental factors shaping the bacterial community compositions in urban green spaces

**Figure 5:**
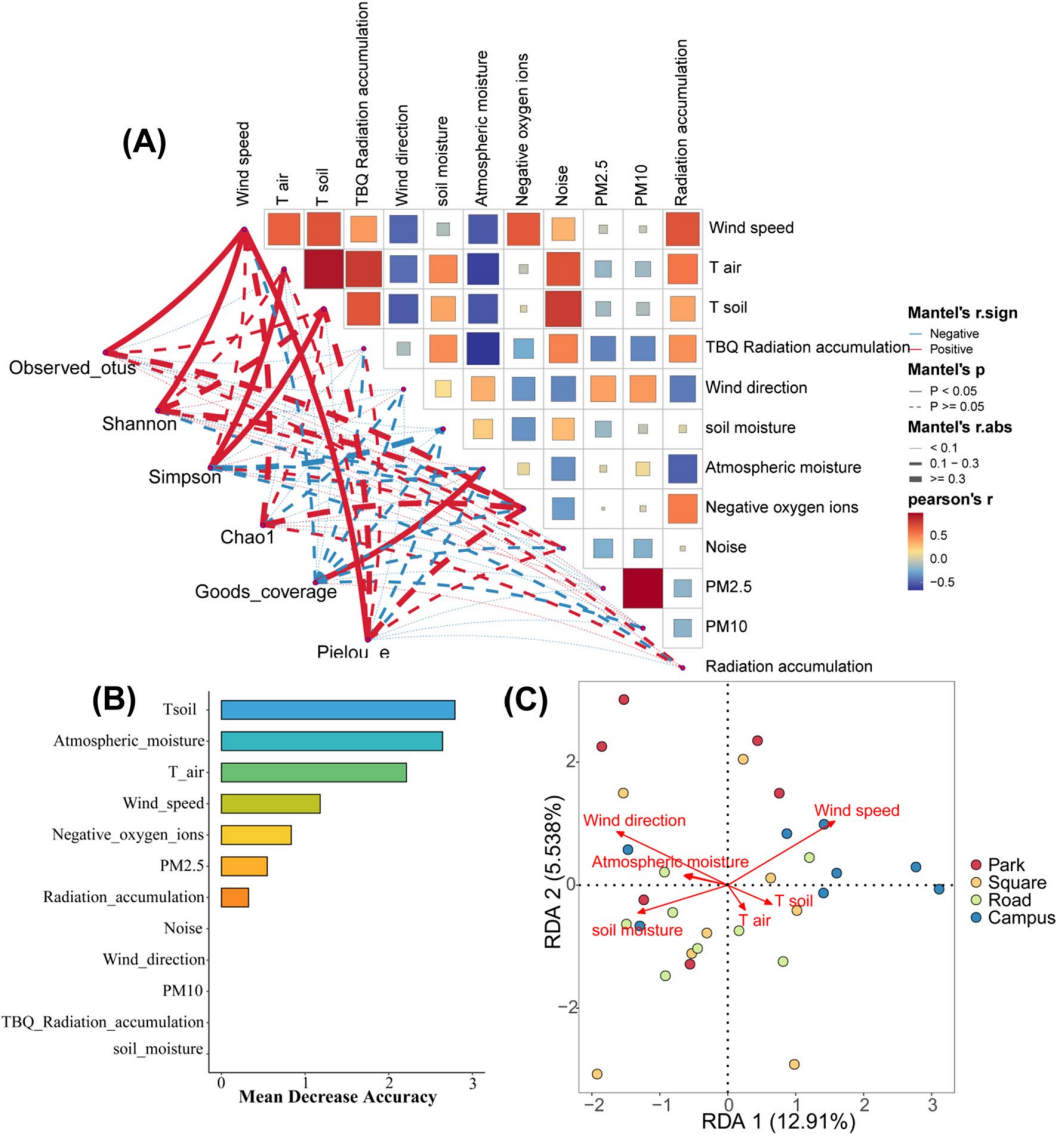
(**A**) A color gradient indicating Pearson’s correlation coefficients was used to depict pairwise comparisons of environmental factors. Partial Mantel tests reveal the relationships between each environmental factor and the diversity indexes. Edge width represents Mantel’s r statistic for the corresponding distance correlations, while edge color indicates the statistical significance. (**B**) Random forest analysis predicts the important influencing factors for bacterial alpha diversity. (**C**) Redundancy analysis (RDA) biplot of the distribution of bacterial communities with environmental factors in green space types.

For the environment properties, mainly the air temperature, soil temperature, TBQ radiation accumulation, and noise correlated positively with wind speed, while wind direction and atmospheric moisture correlated negatively with wind speed, air temperature, soil temperature, and TBQ radiation accumulation (Fig. 5A). The partial Mantel test indicated that the diversity indexes of observed-otu, Shannon, and pielou-e index correlated significantly with wind speed. Simpson index correlated significantly with air temperature and soil temperature, and goods coverage correlated significantly with atmospheric moisture. Most other physicochemical contents were significantly negatively correlated with the diversity indexes. The results of the random forest model showed that the rank order of importance of community environmental factors was soil temperature, atmospheric moisture, air temperature, wind speed, negative oxygen ions, PM2.5, and radiation accumulation (Fig. 5B). The results of RDA showed that the surface bacteria community was regulated by multiple environmental variables (Fig. 5C). The first axis of RDA explained 12.91% of the variation of species-environment relation, while the two axes together explained 18.44% of variation (*p* = 0.001). Wind direction, wind speed, and soil moisture appeared to be the three most significant factors affecting the bacteria community. Wind speed have significant positive association with Campus samples, and wind direction and soil moisture have negative association with campus samples (*p* < 0.05). The relationships between the other samples and environmental parameters were not significant.

### Correlation network analysis between microbial community

**Figure 6:**
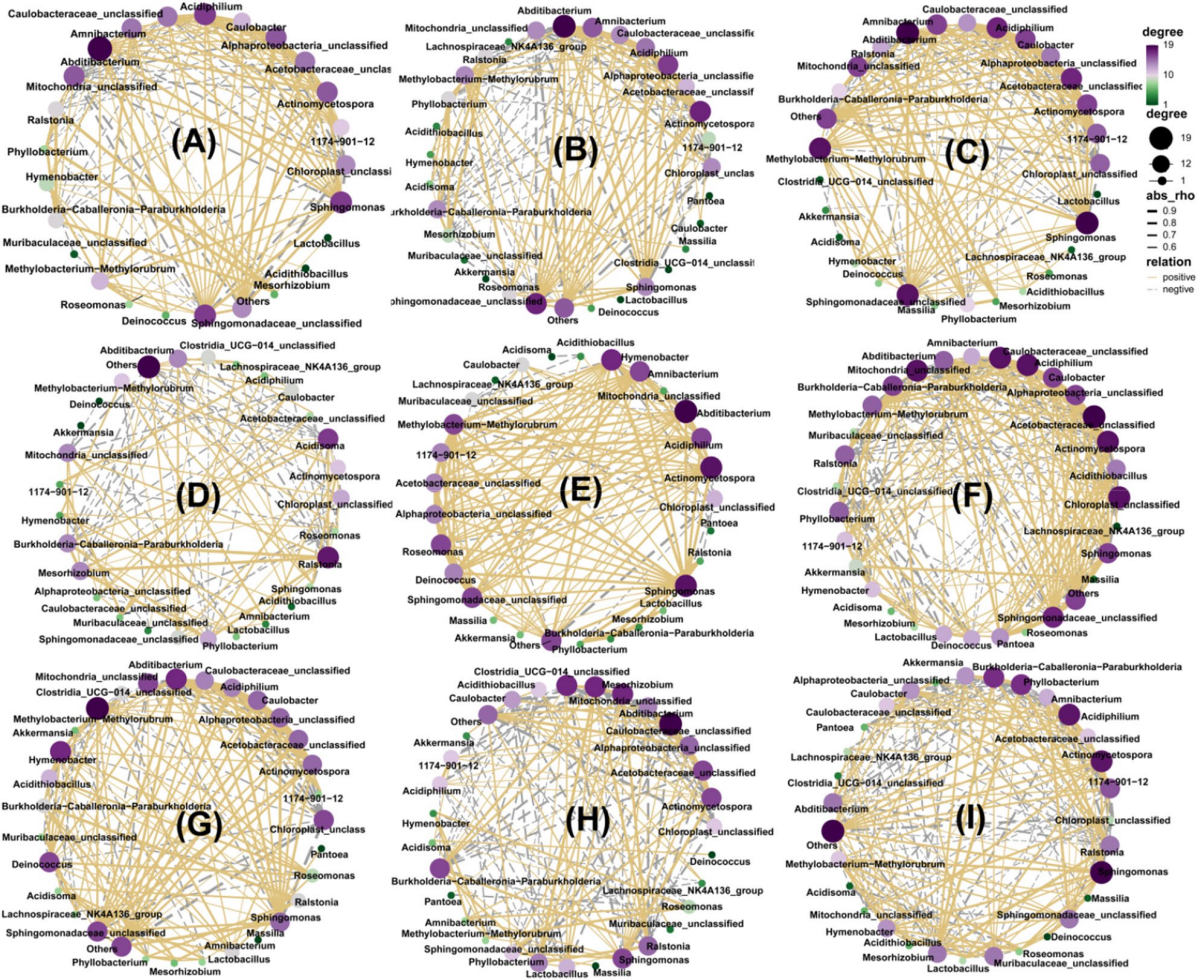
Network complexity of community (**A**) total sample, (**B**) shrub (**C**) tree, (**D**) bark surface, (**E**) leaf surface, (**F**) park, (**G**) square, (**H**) road, (**I**) campus.

The average changes in network properties were used to define network complexity, and average degrees and edges were the most important parameters^40^. Edges indicate significant correlations between microbial taxa, and average degrees indicate the network's overall connectivity. To determine the correlations among species, the richness information at the genus level was used for the top 30 species based on their richness in the different samples. Correlation networks are presented in Fig. 6A–H. There is a correlation between the two species indicated by the connections between the nodes, a positive correlation is shown by the yellow solid line, and a negative correlation is shown by the grey dashed line. A connection's strength is determined by the thickness of the line between any two nodes. The size and color of the dots represent the number of related objects. According to the co-occurrence network analysis of phyllosphere bacteria, the composition of the bacterial community is strongly correlated among whole samples (Fig. 6A, Spearman’s > 0.7, *P* < 0.05, average degree:11.52). *Abditibacterium*, *Sphingomonas*, *Sphingomonadaceae*_unclassified, and *Acidiphilium* dominated the community structure, with node degrees 20, 17, 17 and 17 respectively. Between *Burkholderia-Caballeronia-Paraburkholderia* and *Ralstonia, Mesorhizobium* and *Phyllobacterium*, *Actinomycetospora*, and *Sphingomonas*, there were positive correlations, with rho 0.954, 0.938 and 0.935 respectively. Between Chloroplast_unclassified and others, *Chloroplast*-unclassified and *Sphingomonas*, *Mitochondria*_unclassified and others, there were negative correlations, with rho -0.942, -0.867 and -0.810 respectively. Between the tree and shrub groups, there were large changes in the major species in the community structure (Fig. 6B,C). The co-occurrence network for tree samples showed that the network complexity (edge: 112.501; average degree: 11.310) was greater than the shrub samples (edge: 103.267; average degree: 9.742). *Abditibacterium*, *Sphingomonadaceae*_unclassified, and *Actinomycetospora* dominated the community structure in shrub samples with node degrees 20, 18, and 18, while *Sphingomonas*, *Abditibacterium*, and *Sphingomonadaceae*_unclassified in tree samples with node degree 19, 18 and 18. There were positive correlations between *Methylobacterium-Methylorubrum* and *Roseomonas*, *Mesorhizobium* and *Ralstonia*, and *Actinomycetospora* and *Alphaproteobacteria*_unclassified in shrub samples, with rho 0.952, 0.943 and 0.930 respectively. There were negative correlations between *Chloroplast*_unclassified and *Sphingomonas*, *Chloroplast*_unclassified and Others, and *Mitochondria*_unclassified and *Sphingomonas* in shrub samples, with rho − 0.921, − 0.903 and − 0.886 respectively. In the tree group, there were positive correlations between *Mesorhizobium* and *Phyllobacterium*, *Mesorhizobium* and *Ralstonia*, and *Amnibacterium* and *Methylobacterium*-*Methylorubrum*, with rho 0.962, 0.941 and 0.932 respectively. Negative correlations were presented between *Chloroplast*_unclassified and Others, *Acetobacteraceae*_unclassified and *Chloroplast*_unclassified, and *Chloroplast*_unclassified and others, with rho -0.941, -0.865 and -0.856 respectively. Similarly, on the bark epiphytic and phyllosphere, there were significant differences in the relationships between microorganisms (Fig. 6D,E). On the bark epiphytic, *Ralstonia*, *Acidisoma*, and *Mesorhizobium* were the top three species that dominated the community structure, while *Abditibacterium*, *Sphingomonas,* and *Actinomycetospora* in the phyllosphere. The co-occurrence network for leaf samples showed that the network complexity (edge: 101.126; average degree: 10.000) was greater than bark samples (edge: 71.448; average degree: 7.724). A similar analysis also arose between different green space types (Fig. 6F–I), the network complexity of park green space was greater than the other three green spaces. In the park, square, campus, and road green space types, the distribution of species that account for the most importance in the community was *Acetobacteraceae*_unclassified, *Methylobacterium*-*Methylorubrum*, *Sphingomonas*, *Caulobacteraceae*_unclassified respectively.

## Discussion

### Microbial diversity and community structure in urban green spaces are affected by multiple aspects of urbanization

There are unique opportunities for human and natural environmental microbes to interact on the surface of plants in urban greenspaces, which are vital but neglected ecological milieus. As a result of this study, proteobacteria, cyanobacteria, actinobacteria, and firmicutes were abundant in bacterial communities both in trees and shrubs. These phyla have been found to be abundant in the phyllosphere in previous studies^41,42^. It has also been reported that *Sphingomonas* and *Methylobacterium* (Proteobacteria) are ubiquitously present in the phyllosphere of trees and grasses^43,44^. Further, some Sphingomonas strains can protect plants from plant pathogens, while members of the Methylobacterium can utilize methanol released by plants to promote their host's growth^45,46^. To maintain the productivity and resilience of urban ecosystems, especially under the conditions of global climate change, these taxa should be further explored for their features such as plant growth promotion and biocontrol^47,48^.

A significant impact of plant types and green spaces on alpha and beta diversity of bacteria in bark epiphytic and phyllosphere was observed in this study. Based on the fact that bark and leaves may be the primary sources of airborne microbes, it is reasonable to expect that the microbial communities on the surface of plant bark and leaves are closely related to the air microbiome. There may be a significant role for the mass movement of air in shaping airborne microbiomes^49,50^. In our study, trees had more species richness than shrubs, since trees are less affected by air movement than shrubs, and there was a relationship between the lesser wobbling effect of wind action on surface bacteria. A similar situation can be inferred in the grouping of bark and leaves. Our results indicate that microbial diversity and abundance in the bark group exceeded that of the phyllosphere. This may also be since bark are better fixed than leaves and are less affected by air movement. In addition, the morphological structure of the plant surface is also very important in influencing the attachment of bacteria to the surface^51,52^. Different microenvironments in which epiphytic bacteria live may explain this difference. Specifically, epiphytic bacteria are subjected to selection by their host plant via physiology, leaf morphology, and nutrients and volatile organic compounds (e.g. methanol) exported to the surface epiphytic bacteria, and the surrounding atmosphere, such as solar humidity, radiation, and temperature^47,53,54^. Compared to leaves, bark epiphytic have better conditions for providing attachment of microorganisms such as bacteria, which is consistent with our analyses that bark epiphytic retain more species abundance and complex community composition. Some studies have shown that some interleaf microorganisms have important functions such as promoting plant growth, adsorbing and degrading environmental pollutants, and guiding the closure of leaf stomata to prevent the entry of pathogenic bacteria^55^. Plants play an important role in phosphate dissolution, nitrogen fixation, nitrification, and balancing the global carbon and nitrogen cycles^56^. Plant identity significantly affected both the diversity and community structure of phyllosphere epiphytic and endophytic bacteria, as previous studies have shown^44,57,58^. Various plant species possess various functional traits, such as specific leaf area, nutrient content, osmotic properties, respiration rate, and dust retention efficiency which are significantly correlated with the surface bacteria community of leaf and stem^59–61^. In various samples of UGS’s type, microbial communities exhibited inherent differences, possibly due to their inherent adaptability. Consequently, nutrient disturbances led to a decline in nutrient-tolerant bacteria, and they were then replaced by generalists^62^. There is generally an increase in species richness with the area as illustrated by the species-area relationship^63^, and larger area urban green spaces are less exposed to urbanization and human impacts, and the microbiologic community is relatively less adversely affected. Of our different green space types, campuses and parks have larger areas, however in terms of OTU abundance, parks and roads go on to have greater abundance.

### Various environmental factors as an important factor shaping the bacterial communities in urban green spaces

We characterized the relationships between leaf and bark phyllosphere bacterial community compositions in urban green spaces with a variety of factors including soil, air, noise, and radiation properties. Some soil properties (soil pH, soil moisture, and soil texture ) are key drivers in shaping soil microbial communities have been shown in many studies^64,65^, and in this study, surprisingly, soil properties remained also one of the most important factors in shaping the characteristics of bacterial communities on the phyllosphere and bark epiphytic. In addition, air environmental parameters were second only to soil environmental parameters as important microbial community drivers analyzed by random forest. Some studies have reported that the more highly urbanized a place is, the more homogenous the community structure of plants^66^, ground animals^67^, birds^68^, and insects^69^. In our study, environmental factors that characterize urbanization were correlated with microbial diversity and also showed that the main factors of the urban greenhouse effect (air and soil temperature and humidity) were significantly correlated with microbial diversity. Species diversity is significantly and positively correlated with both community structure and stability^70^, therefore, the greenhouse effect of urbanization can significantly affect the structure of bacterial communities on the surface of plant bark and leaves. In the RDA analysis, wind speed and direction were very important influences, which were very relevant to the effect of physical air movement on microbial attachment discussed above. Current research lacks in-depth linkages between environmental factors and microbial function, as well as the relationship between microbial function and human health, and future in-depth studies through large-scale and longitudinal analyses may lead to a better understanding of the microbial mechanisms underlying the impact of urban green spaces on human health.

In this study, we analyzed the differences in community structure and interspecific differences among different groups by correlation network analysis. Overall, trees have a more complex network structure than shrubs, and leaves have a more complex network structure than bark. An result here is that leaves have less microbial abundance relative to bark, but a complex community structure correlation. Plants' leaves are important organs determining their response to environmental change and highly plastic in long-term evolution^71^, converting energy in ecosystems^72^. Aside from that, leaves are essential for photosynthesis and nutrient uptake in plants, so their role in plant growth is evident^73^. Since plant leaves grow and fall off periodically and are greatly affected by factors such as external air, it is possible to propose the idea that although leaf microbes are not as abundant as bark, they need to be more cohesive in order to keep the leaves growing and to maintain important physiological functions. It has been shown that plants indicate significant differences in microbial communities between urban habitats and that such differences are largely dependent on plant species^74^. In our results, the highest abundance of attached microorganisms and stronger community associations were found in parkland green spaces relative to several other green space types. Some studies have shown that ficus is the most suitable plant species for sustainable urban planting because of its high dust deposition^75^. It can be inferred that *Ficus virens*, the dominant tree in the parkland in this study, can play an important contribution by adsorbing microorganisms through dust retention. According to analysis of correlation network, *Abditibacterium*, *Sphingomonas*, *Sphingomonadaceae*_unclassified, and *Acidiphilium* dominated the whole community structure, which not dominant in high abundance. This revealed that the keystone taxa were not mostly observed in abundant species, which may be essential for preserving the ecosystem’s functions. The present study lacks linkage to microbial function, and future indepth studies with large scale and longitudinal analyses to identify phyllosphere and stem microbiomes of different urban green spaces may provide better insight into the microbial mechanisms underlying urban greenspaces impact on human health.

## Conclusions

This work investigated the microbial communities of phyllosphere and stem epiphytes and Influence of climatic factors with diverse UGS types. Our results revealed that urban greenspaces'type altered the way communities gathered. There also was significant α-diversityin between bark epiphytic and phyllosphere community.The main soil and air properties factors of the urban greenhouse (e.g. soil temperature, atmospheric moisture, air temperature) were shaping the characteristics of bacterial communities. network analysis revealed keystone taxa were not mostly observed in abundant species, which may be necessary to maintain ecosystem functions. Consequently, this research expands our comprehension of the multifaceted ecological functions fulfilled by UGS, shedding new light on their intricate role in microbial ecology.

### Supplementary Information

Supplementary Table S1.

## Data Availability

The sequence data presented in the study were deposited in the Sequence Read Archive (SRA) repository of NCBI at https://www.ncbi.nlm.nih.gov/sra, accession number PRJNA1097078. The environmental parameters datasets used or analysed during the current study available from the corresponding author on reasonable request.
